# Neutrophil-to-lymphocyte ratio is a prognostic factor for colon cancer: a propensity score analysis

**DOI:** 10.1186/s12885-020-07429-5

**Published:** 2020-09-25

**Authors:** Junichi Mazaki, Kenji Katsumata, Kenta Kasahara, Tomoya Tago, Takahiro Wada, Hiroshi Kuwabara, Masanobu Enomoto, Tetsuo Ishizaki, Yuichi Nagakawa, Akihiko Tsuchida

**Affiliations:** Department of Gastrointestional and Pediatric Surgery, Tokyo Medical University, Tokyo, Japan

**Keywords:** Neutrophil-to-lymphocyte ratio (NLR), Relapse-free survival, Overall survival, Propensity score, Propensity score matching, Colon cancer, Sidedness

## Abstract

**Background:**

A large number of patients suffer recurrence after curative resection, and mortality from colon cancer remains high. The role of systemic inflammatory response, as reflected by neutrophil-to-lymphocyte ratio (NLR), in cancer recurrence and death has been increasingly recognized. This study aimed to analyze long-term oncologic outcomes of Stage II-III colon cancer to examine the prognostic value of NLR using a propensity score analysis.

**Methods:**

A total of 375 patients with colon cancer underwent radical surgery between 2000 and 2014 at Tokyo Medical University Hospital. Long-term oncologic outcomes of these patients were evaluated according to NLR values. A cut-off NLR of 3.0 was used based on receiver operating characteristic curve analysis. Primary outcomes were overall survival (OS) and relapse-free survival (RFS). An analysis of outcomes according to tumor sidedness was also performed.

**Results:**

Patients with lower NLR values (“lower NLR group”) were more likely to have lymph node metastasis compared to those with higher NLR values (“higher NLR group”) before case matching. After case matching, clinical outcomes were similar between the two groups. There were no significant differences in 5-year OS and 5-year RFS rates between the two groups before case matching based on propensity scores. After case matching, 5-year OS rates were 94.5% in the lower NLR group (*n* = 135) and 87.0% in the higher NLR group (n = 135), showing a significant difference (*p* = 0.042). Five-year RFS rates were 87.8% in the lower NLR group and 77.9% in the higher NLR group, also showing a significant difference (*p* = 0.032). Among patients with left-sided colon cancer in the matched cohort, 5-year OS and 5-year RFS rates were 95.2 and 87.3% in the lower NLR group (*n* = 88), respectively, and 86.4 and 79.2% in the higher NLR group (*n* = 71), respectively, showing significant differences (*p* = 0.014 and *p* = 0.047, respectively).

**Conclusions:**

The NLR is an important prognostic factor for advanced colon cancer, especially for left-sided colon cancer.

## Background

Treatment strategies for colon cancer are well-established, and include surgical resection and chemotherapy. However, many patients suffer recurrence after curative surgical resection, and mortality rates from colon cancer remain high. The role of systemic inflammatory response in cancer recurrence and death has been increasingly recognized [1]. In addition to tumor characteristics, the host immune system plays an important role in the development of colon cancer [2]. Many immunological and nutritional markers have been reported to be prognostic factors for different types of cancer, including colon cancer [3–7]. Among these, neutrophil-to-lymphocyte ratio (NLR) is calculated from white blood cell (WBC) differential counts and can be obtained easily in preoperative patients. Previous studies have reported on the predictive potential of NLR as a prognostic factor in various types of malignancies, including resectable colon cancer [8–14]. However, since these previous studies analyzed data by univariate or multivariate analysis, the possibility of selection bias and confounding factors could not be ruled out. Moreover and up to our knowledge, no prospective cohort studies have been conducted. To address these limitations and to increase the strength of evidence, two studies used a propensity score analysis [15, 16]. However, one of these studies had a small sample size (*n* = 200) and included patients of all disease stages (Stage I-IV), while the other study targeted only those with early-stage colon cancer.

The present study aimed to analyze long-term oncologic outcomes of patients with Stage II-III colon cancer at our institute to examine the prognostic value of NLR using a propensity score analysis.

## Methods

### Patients

Medical records of 422 patients who underwent radical surgery for Stage II-III colon cancer between 2000 and 2014 at Tokyo Medical University Hospital were retrospectively reviewed. Of these, 47 patients without NLR values were excluded, and the remaining 375 patients were divided into two groups based on NLR values (lower NLR and higher NLR groups). In general, patients were admitted to our hospital two days before operation, and NLR values were obtained on the day of admission. According to receiver operating characteristic (ROC) curve analyses and previous reports, a cut-off value of 3.0 was set for both mortality and recurrence (mortality: area under the curve (AUC) = 0.521, 95%CI 0.449–0.592; recurrence: AUC = 0.502, 95%CI 0.423–0.581). This study was approved by the institutional review board of Tokyo Medical University Hospital.

### Surgical treatment

All patients underwent curative surgery. Gross specimens were opened up along the antimesenteric border, and lymph nodes were harvested from the mesocolon. The specimens were then laid out on a board and fixed in 10% formalin.

### Postoperative systemic adjuvant chemotherapy

Postoperative systemic adjuvant chemotherapy is generally performed for pStage III colon cancer at our institute. In the present study, adjuvant chemotherapy was performed in 19 of 216 patients (8.8%) with pStage II disease and 86 of 202 (42.5%) patients with pStage III disease. Oxaliplatin-based and 5-fluorouracil-based regimens were most commonly used.

### Follow-up

Median follow-up period was 73.2 months (range, 0.2–225.1 months). For follow-up, patients with Stage II or III tumors were examined for up to five years postoperatively. Specifically, tumor marker measurements were performed every three months for the first two years, and both tumor marker measurements and CT scans were performed every six months for the next three years. When the first recurrence occurred, the recurrence site and date were recorded.

### Statistical analysis

Primary outcomes were 5-year overall survival (OS) and 5-year relapse-free survival (RFS). OS was defined as the interval between the date of operation and the date of either death or the end of the observation period. Patients alive at the end of follow-up were censored. RFS was defined as the interval between the date of operation to date of recurrence, or death from underlying disease. Observations were censored when patients died for a reason other than colon cancer. Survival characteristics were assessed using the Kaplan-Meier method and were compared using the log-rank test. Propensity score analyses were performed to adjust for heterogeneity between two groups by NLR. Multivariate logistic regression was used to generate a propensity score predicting condition by NLR (NLR > 3.0 or NLR ≤ 3.0). The following six covariates were included: age, sex, body mass index (BMI), tumor size, pathological T-stage, and pathological lymph node metastasis. Each patient was assigned an estimated propensity score, which represented the patient’s predicted probability of the NLR status. We specified matching IDs based on the propensity scores. According to the matching IDs, we divided the patients into two groups by their NLR values, in which patients were paired by similarities in their characteristics. Then, propensity score matching was performed. Each patient with a NLR < 3.0 (lower NLR) was matched to a patient with a NLR ≥ 3.0 (higher NLR) and had the closed propensity score on the logit scale with a caliper of 0.05. Standardized mean difference (SMD) was calculated to evaluate variable balance after propensity matching. Propensity scores were used for regression adjustment, in which the treatment effect was estimated by adjusting for the impact of background covariates in a regression model. We also performed analyses by dividing patients into two groups according to tumor sidedness (right-side colon: from cecum to transverse colon; left-side colon: from splenic flexure to rectosigmoid colon). All statistical analyses were performed using SPSS software (IBM® SPSS® Statistics for Windows, Version 25.0; IBM, Chicago, IL, USA). The level for statistical significance was set at *p* < 0.05.

## Results

### NLR

The median NLR was 2.5 (range, 0.2–56.2) for the entire cohort (*n* = 375). There were 230 patients in the lower NLR group and 145 patients in the higher NLR group. The median NLR was 3.0 (range, 0.4–56.2) after case matching (*n* = 270 and 135 in each group, respectively).

### Patient and tumor characteristics

Baseline characteristics are summarized in **Table** **1**. Patients in the lower NLR group had significantly higher rates of pathological lymph node metastasis compared to those in the higher NLR group (42.5% vs 52.5%, *p* = 0.006). No significant differences were observed in other covariates.

**Table 1.**
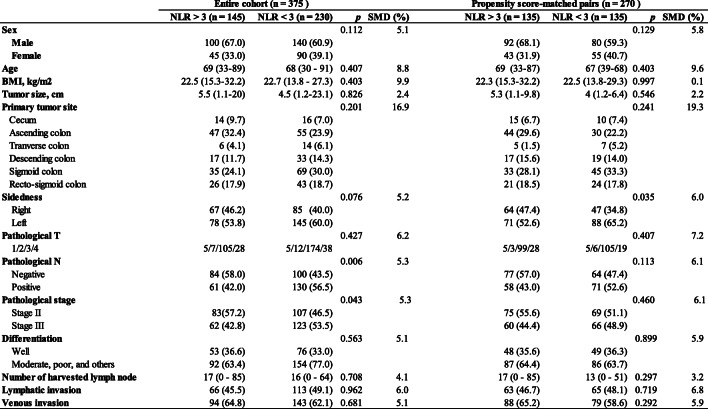
Baseline characteristics.

Data are expressed as median (range) or n (%)

*NLR* Neutrophil-to-Lymphocyte Ratio, *BMI* Body Mass Index, *SMD* Standardized Mean Difference

*Others included mucinous adenocarcinoma and papillary adenocarcinoma

**Fig. 1:**
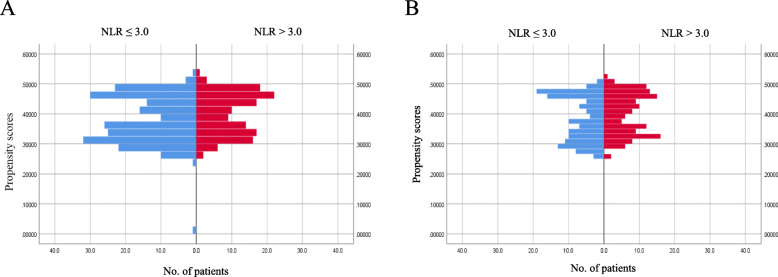
Distributions of propensity scores before and after case matching. **a** Distribution of propensity scores in the entire cohort. b Distribution of propensity scores in the propensity score-matched cohort

A matched analysis was performed according to propensity scores to adjust for heterogeneity in the lower NLR and higher NLR groups, with six covariates (i.e., age, sex, BMI, tumor size, pathological T-stage, and pathological lymph node metastasis). Distributions of propensity scores before and after case matching are shown in Fig. 1a and b. Both lower NLR and higher NLR groups (135 matched pairs) showed a well-matched distribution with respect to patient and tumor characteristics in the adjusted analysis after case matching (Table 1). No significant difference was observed between the two groups in pathological lymph node metastasis rate.

### Regression adjustment including propensity scores

Cox models were created by applying propensity scores to adjust for group differences via regression adjustment. In the entire cohort (*n* = 375), hazard ratios (HRs) for OS and RFS in NLR > 3.0 versus NLR < 3.0 were 1.546 (95%CI 0.879–2.718) and 1.419 (95%CI 0.910–2.215), respectively (Tables 2, and 3).

**Table 2.**
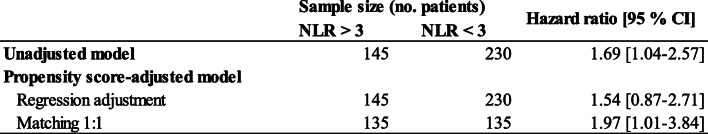
Hazard ratios for overall survival to measure the effects of NLR

*NLR* Neutrophil-to-Lymphocyte Ratio

**Table 3.**
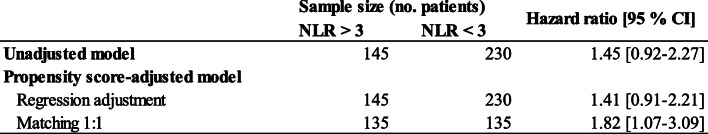
Hazard ratios for recurrence free survival to measure the effects of NLR

*NLR* Neutrophil-to-Lymphocyte Ratio

### OS and RFS rates

**Fig. 2:**
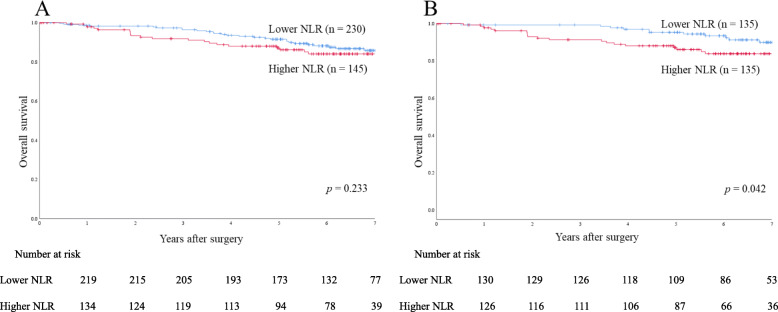
Overall survival rates in the entire cohort and propensity score-matched cohort. **a** Overall survival rates in the entire cohort (*n* = 375). **b** Overall survival rates in the propensity score-matched cohort (135 matched pairs)

**Fig. 3:**
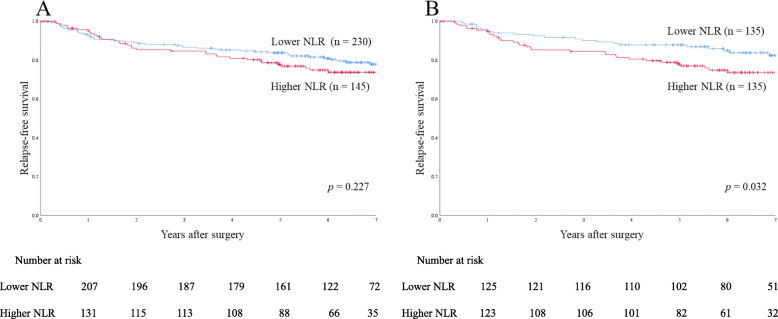
Relapse-free survival rates in the entire cohort and propensity score-matched cohort. **a** Relapse-free survival rates in the entire cohort (n = 375). **b** Relapse-free survival rates in the propensity score-matched cohort (135 matched pairs)

In the entire cohort, 5-year OS and 5-year RFS rates were 90.1 and 81.7%, respectively. Before case matching, 5-year OS and 5-year RFS rates were 91.5 and 83.7% in the lower NLR group (*n* = 230), respectively, and 87.1 and 77.7% in the higher NLR group (*n* = 145), respectively, with no significant differences (*p* = 0.233 and *p* = 0.227, respectively) (Figs. 2a and 3a).

The analysis of survival data after case matching revealed a significant difference in 5-year OS rate between the lower NLR group (*n* = 135; 94.5%) and the higher NLR group (n = 135; 87.0%) (*p* = 0.042) **(**Fig. 2b**)**. A significant difference was also observed in 5-year RFS rate after case matching betw0een the lower NLR group (87.8%) and the higher NLR group (77.9%) (*p* = 0.032) **(**Fig. 3b**)**.

**Fig. 4:**
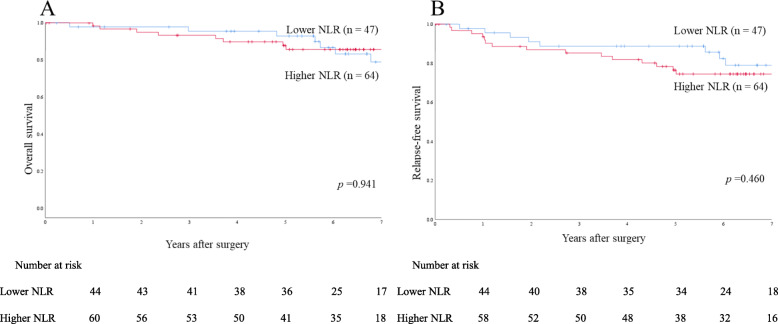
Survival rates in the propensity score-matched cohort of right-sided colon cancer patients. **a** Overall survival. **b** Relapse-free survival

**Fig. 5:**
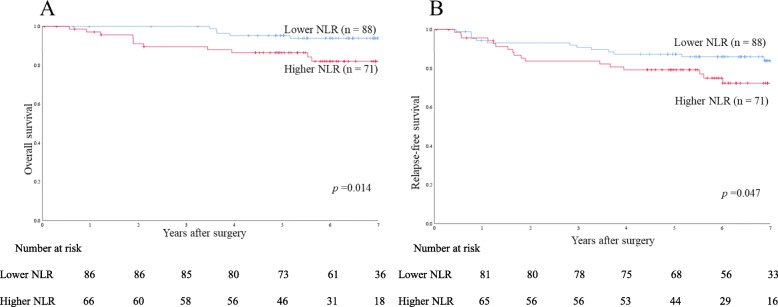
Survival rates in the propensity score-matched cohort of left-sided colon cancer patients. **a** Overall survival. **b** Relapse-free survival

### Tumor sidedness

To evaluate the prognostic value of NLR by tumor sidedness, patients were divided into two groups according to tumor location in the colon. Survival data were analyzed for case-matched patients. Among patients with right-sided colon cancer, 5-year OS and 5-year RFS rates were 92.9 and 88.8% in the lower NLR group (*n* = 47), respectively, and 87.8 and 76.5% in the higher NLR group (*n* = 64), respectively, showing no significant difference (*p* = 0.941 and *p* = 0.460, respectively) (Figs. 4 a and 5 a). Among patients with left-sided colon cancer, 5-year OS and 5-year RFS rates were 95.2 and 87.3% in the lower NLR group (*n* = 88), respectively, and 86.4 and 79.2% in the higher NLR group (*n* = 71), respectively, showing significant differences (*p* = 0.014 and *p* = 0.047, respectively) (Figs. 4b and 5b).

## Discussion

Colorectal cancer is the second leading cause of cancer death in Japan. Since local recurrence and distant metastasis occur in a large number of patients even after curative surgery, a new focus has been placed on identifying biomarkers that predict prognosis. While tumor-related factors have been investigated in many previous studies, host-related factors have only recently started to draw attention [1].

The host immune system is an important factor which affects the outcome of cancer [16–18]. NLR has been suggested to be a simple index of systemic inflammatory response. Neutrophilia occurs during systemic inflammation, and lymphopenia is a maker for depressed cell-mediated immunity [19]. That is, cell-mediated immune responses are dependent largely on lymphocytes. A large number of lymphocytes at tumor sites has been shown be associated with a good prognosis, whereas lymphopenia has been reported to be a predictor of poor prognosis [20]. On the contrary, neutrophils suppress lymphocyte-mediated cytolysis and has been reported to be associated with a poor prognosis [21]. The prognostic NLR in malignancy may due to the high tumor angiogenesis activity of tumor-induced neutrophils contributing to tumor progression, lymphocyte count associated with disease severity, and immune escape of tumor cells from tumor infiltrating lymphocyte [22].

Walsh et al. first reported a correlation between preoperatively elevated NLR had a relationship with overall and cancer-specific survival in colon cancer [23]. The predictive potential of NLR in evaluating the prognosis of patients with various types of malignancies, including resectable colon cancer, has been demonstrated in previous studies [8–11]. However, no study has analyzed a large cohort of patients with Stage II-III advanced colon cancer using propensity scores to minimize selection bias. The present study cohort consisted of 375 patients with Stage II-III colon cancer, and no significant differences were observed between the lower NLR and higher NLR groups in terms of OS and RFS before case matching. After case matching according to propensity scores, however, NLR was found to predict prognosis both in terms of OS and RFS with a cut-off value of 3.0.

The colon originates from the midgut and hindgut during embryonic development and differentiates into the right-side colon and left-side colon. Tumors in the cecum, ascending colon, and the proximal part of the transverse colon are defined as midgut tumors, and those in the distal transverse, descending, and sigmoid colon and the rectum are defined as hindgut tumors. Differences have been reported between right and left-sided colon tumors in terms of clinical symptoms, incidence, molecular pathways involved, and oncologic outcomes, as well as embryologic origin [24–28]. However, no reports have examined the predictive potential of NLR in colon cancer with a focus on tumor sidedness. The present study evaluated the prognostic value of NLR by tumor sidedness, and found that both 5-year OS and 5-year RFS rates were significantly lower in patients with left-sided colon cancer who had a higher NLR. In contrast, no significant differences were observed among patients with right-sided colon cancer.

National Comprehensive Cancer Network (NCCN) guidelines recommend adjuvant chemotherapy for high-risk Stage II and Stage III colon cancer [29]. High-risk factors for recurrence include poorly differentiated histology, lymphatic/vascular invasion, bowel obstruction, < 12 lymph nodes examined, perineural invasion, localized perforation, and close, indeterminate, or positive margins. The guidelines, however, also state that there are no data correlating risk features with selection of chemotherapy. In the present study, preoperative NLR was associated with both RFS and OS, suggesting that NLR could be used as a tool to identify patients for whom adjuvant chemotherapy should be performed/avoided, especially for left-sided colon cancer. A prospective study will be needed to further explore this possibility.

This study has some limitations. First, we used a single-center retrospective design. However, to minimize selection bias, we analyzed the data according to propensity scores. Second, our data did not include records of whether patients had hematologic or autoimmune disease, which may have influenced preoperative NLR values. Third, no molecular assessment was performed in this study, e.g., to determine microsatellite instability. A prospective study will be needed to examine confounders and further clarify the prognostic value of NLR.

## Conclusions

The present study demonstrated that NLR could be used as a prognostic factor for advanced colon cancer, especially for left-sided colon cancer.

## Data Availability

The datasets used and/or analyzed during the current study are available from the corresponding author on reasonable request.

## Abbreviations

NLR: Neutrophil-to-Lymphocyte Ratio
BMI: Body Mass Index
OS: Overall Survival
RFS: Relapse-Free Survival
AUC: Area Under the Curve
